# Lysine methylation of HIV-1 Tat regulates transcriptional activity of the viral LTR

**DOI:** 10.1186/1742-4690-5-40

**Published:** 2008-05-22

**Authors:** Rachel Van Duyne, Rebecca Easley, Weilin Wu, Reem Berro, Caitlin Pedati, Zachary Klase, Kylene Kehn-Hall, Elizabeth K Flynn, David E Symer, Fatah Kashanchi

**Affiliations:** 1 The George Washington University Medical Center, Department of Biochemistry and Molecular Biology, Washington, DC 20037, USA; 2 Basic Research Laboratory, Center for Cancer Research, National Cancer Institute, Frederick, MD 21702, USA; 3 Basic Research Laboratory, and Laboratory of Biochemistry and Molecular Biology, Center for Cancer Research, National Cancer Institute, Frederick, MD 21702, USA; 4 W.M. Keck Institute for Proteomics Technology and Applications, Washington, DC 20037, USA

## Abstract

**Background:**

The rate of transcription of the HIV-1 viral genome is mediated by the interaction of the viral protein Tat with the LTR and other transcriptional machinery. These specific interactions can be affected by the state of post-translational modifications on Tat. Previously, we have shown that Tat can be phosphorylated and acetylated *in vivo *resulting in an increase in the rate of transcription. In the present study, we investigated whether Tat could be methylated on lysine residues, specifically on lysine 50 and 51, and whether this modification resulted in a decrease of viral transcription from the LTR.

**Results:**

We analyzed the association of Tat with histone methyltransferases of the SUV39-family of SET domain containing proteins *in vitro*. Tat was found to associate with both SETDB1 and SETDB2, two enzymes which exhibit methyltransferase activity. siRNA against SETDB1 transfected into cell systems with both transient and integrated LTR reporter genes resulted in an increase in transcription of the HIV-LTR in the presence of suboptimal levels of Tat. *In vitro *methylation assays with Tat peptides containing point mutations at lysines 50 and 51 showed an increased incorporation of methyl groups on lysine 51, however, both residues indicated susceptibility for methylation.

**Conclusion:**

The association of Tat with histone methyltransferases and the ability for Tat to be methylated suggests an interesting mechanism of transcriptional regulation through the recruitment of chromatin remodeling proteins to the HIV-1 promoter.

## Background

The HIV-1 genome incorporates nine viral genes, all of which are expressed from a single promoter located within the viral long terminal repeat (LTR) [1,2]. The activity of the HIV-1 promoter is strongly dependant on the viral transactivator, Tat, the protein responsible for transcriptional activation and elongation [3-8]. The main function of Tat is to activate the HIV-1 LTR by binding to an RNA stem-loop structure, TAR [3,4,6,9-11]. This interaction initiates a binding cascade where cellular transcription factors such as Cdk9 and cyclin T1 are recruited to the HIV-1 promoter to facilitate viral transcription [12-15]. Tat mediates the functional modifications associated with viral transcription primarily by interacting with host cellular kinases, specifically to phosphorylate the large subunit of RNA Pol II CTD resulting in the activation of elongation [12,16,17]. In addition to the recruitment of host cellular proteins and enzymes for transcriptional initiation, such as NF-κB, Sp1, and TFIID, Tat has also been shown to bind a number of other factors which regulate chromatin structure located at the HIV promoter thus allowing access to the LTR DNA [9,10,18-27].

The basic building blocks of chromatin are organized into nucleosomes, each of which is made up of 146 bp of DNA wrapped around an octamer of histone proteins that consists of two copies of each of H2A, H2B, H3, and H4. The nucleosome can be divided into two domains, one of which is the structured histone-DNA and histone-histone globular domain, and the other is the highly basic N-terminal histone tails which contain multiple sites for post-translational modifications including acetylation, phosphorylation, methylation, ubiquitination, and sumoylation [28-31]. The post-translational modifications present on each histone tail can direct higher order chromatin structure and consequently, transcription through a cycle of conflicting activation and repression signals [32-34]. Histone acetyltransferases (HATs), histone deacetylases (HDACs), kinases, and histone methyltransferases (HMTs) are all responsible for the addition/removal of covalent modifications on the histone tails [35-37]. In the case of retroviruses, the integration of proviral DNA into the genome of an infected cell requires the manipulation of cellular transcriptional machinery as well as cellular chromatin remodelers to accomplish proliferation, replication, and latent infection of the virus. Transcriptional silencing of the HIV-1 genome may be directly correlated with the state of chromatin packaging near the viral integration site [38-40].

Histone methyltransferases (HMTs) can methylate arginine residues such as 2, 8, 17, and 26 on H3 and residue 3 on H4. HMTs can also methylate specific lysine residues such as 4, 9, 27, 36, and 79 on H3 and residue 20 on H4 which serve as markers for the recruitment of chromatin organization complexes [41-43]. Specifically, lysine methylation is catalyzed by the SET-domain family of proteins which function to transfer a methyl group from *S*-adenosyl-L-methionine to the amino group of the lysine side chain, often on lysine 9 of H3 (H3-K9) [41]. Historically, the methylation of H3-K9 has been linked to functionally repressed chromatin [33,44,45]. The selective methylation of H3-K9 results in the recruitment of the HP1 family of heterochromatic binding proteins therefore distinguishing transcriptionally silent chromatin regions [28,33,35,44,46-49]. The SET domain is comprised of approximately 130 amino acids surrounded by other domains which confer substrate specificity. The SUV39 family of SET-domain containing proteins, SUV39H1, SUV39H2, G9a, EHMT1, SETDB1, SETDB2, and SETMAR, specifically methylate lysines on Histone H3, however, more recent studies have also shown a preference for other proteins in addition to histones, therefore lending this family the name of protein lysine methyltransferases [41,50,51].

Lysine is a ~129 Da basic amino acid which is subject to multiple post-translational modifications such as acetylation, methylation, ubiquitination, and sumoylation. Lysine residues contain an ε-amino group which is highly catalytic for many metabolic and chemical reactions. Specifically, lysine residues can be mono-, di-, or trimethylated, each of which can differentially regulate chromatin structure and transcription. The chemical structure of lysine allows for only one type of post-translational modification to be present at any time, also allowing for steric hindrance of the modifications. This system of modification results in the need for both methylases and demethylases in response to particular cellular events. Of particular interest, while a lysine contains a methyl group, it cannot be simultaneously acetylated, therefore resulting in either an "on" or "off" orientation of the molecule. This consequence of the addition of a modification is important when regulating transcriptional activation or repression.

Tat itself is also subject to various post-translational modifications by host cellular proteins. Tat is phosphorylated, acetylated at lysines 28, 50, and 51, ubiquitinated at lysine 71, and methylated at arginine residues 52 and 53 [52-54]. Specifically, the basic domain (residues 49–57), which confers TAR RNA binding, is highly conserved and subject to acetylation on residues K28, K50, and K51 by CBP/p300, the result of which is crucial for Tat transactivation [55-59]. The acetylation of these residues is of great interest as a target for inhibition therapies; the prevention of acetylation would ensure only a low level of viral DNA is transcribed. Also, Tat retains its ability to dynamically shape the foundation of viral transcription through host machinery via its involvement with host cellular kinases. Recent studies have shown that Tat can be methylated by protein arginine methyltransferases (PRMTs) on arginine residues 52 and 53, resulting in a decreased interaction with TAR and cyclin T1 complex formation, therefore decreasing HIV-1 transcriptional activation [54,60]. Here we investigated the methylation of lysine residues 50 and 51, which would compete with and therefore prevent the acetylation of the same residues and any subsequent viral transactivation. We especially were interested in these lines of investigation, since we had previously observed the presence of TIF-1α (a DNA-binding chromatin remodeling protein) when using proteomic analysis to identify cellular proteins bound to unmodified Tat [31]. Here, we report the specific methylation of Tat lysine residues 50 and 51 by protein lysine methyltransferases. Initial screenings of the members of the SET-family for specific interactions with Tat *in vitro *revealed SETDB1/2 to be substrate specific for Tat. We observed that the H3-K9 methyltransferase SETDB1 can specifically methylate Tat preferentially at lysine 51. SiRNA knockdown studies of SETDB1 in transient transfected cells or cells with an integrated LTR reporter gene and associated cellular factors indicated an increase in LTR transactivation in the absence of the inhibitory modification. Collectively, our results imply that the modification of Tat at lysine 51 may contribute to an "on" or "off" phenotype of the HIV-1 promoter.

## Results

### Lysine residue methylation of Tat by histone methyltransferases

The core histone tails have long been a primary example of the importance of post-translational modifications in transcriptional activation and repression. Histone modifications control the higher order chromatin structure and are facilitated by enzymes such as HATs, HDACs, and HMTs. Various combinations of modifications can be involved in the recruitment of specific transcription factors, therefore suggesting the "histone code" hypothesis. Many specific residues of the core histone tails have been identified as integral to transcriptional activation and repression and, consequently, their modifications have been documented. For instance, integral residues such as H3K9, H3K18, and H3K27 can be both acetylated and methylated, however, not simultaneously. Lysine methylation of histones is carried out by the SET-domain containing enzymes; therefore, this family of proteins was subjected to further investigation in the current manuscript.

### Tat associates with SETDB1 and SETDB2 *in vitro*

**Figure 1:**
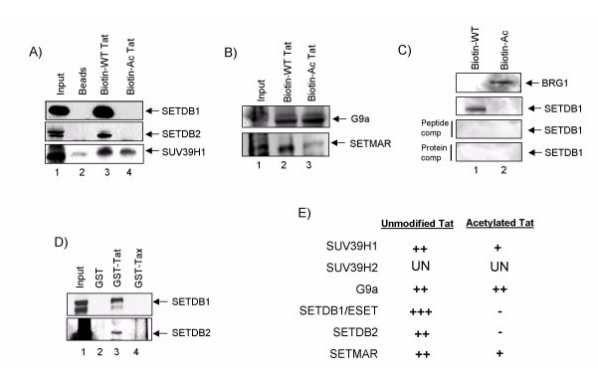
**The co-precipitation of Tat with SET-domain containing proteins**. **A) **Biotin-labeled wild type Tat (Lane 3) and acetylated (residues 50 and 51) Tat (Lane 4) peptide immunoprecipitated complexes were probed for the presence of SETDB1, SETDB2, and SUV39H1. 1/20 of input was used as positive control for western blots. **B) **Biotin-labeled wild type Tat (Lane 2) and acetylated Tat (Lane 3) peptide complexes were probed for the presence of bound G9a and SETMAR. **C) **Positive control reaction using BRG1 pulldown for the acetylated Tat [61], and competition experiment with Tat 42–51 peptide (1:10 ratio) as well as purified wild type Tat 1–86 (1:10 ratio) to compete out SETDB1 binding. **D) **GST-bound wild type Tat and wild type Tax protein complexes were probed for the presence of bound SETDB1 and SETDB2. **E) **A summary of the Tat binding interactions between all members of the SUV39 family as predicted by SMART) [73]. Under both the Unmodified Tat and Acetylated Tat binding affinity column, a "-" indicates that the enzyme does not bind to the indicated form of Tat, while increasing amounts of "+" indicates that the enzyme bound to the indicated form of Tat with a greater specificity. The "UN" indicates that binding affinities were undetermined.

The SUV39 family of SET-domain containing proteins, SUV39H1, SUV39H2, G9a, EHMT1, SETDB1, SETDB2 (unpublished data), and SETMAR, specifically methylate lysine residues of Histone H3, but have also recently been referred to as general protein lysine methyltransferases. We investigated the association of Tat with these enzymes *in vitro*. EHMT1 was excluded from our studies as it is a *Drosophila *analog. SUV39H2 was investigated; however no consistant positive results were seen across immunoprecipitations (undetermined, data not shown). We pulled down protein complexes bound to purified forms of Tat peptides and performed Western blots against each of the above methyltransferases. Purified wild type Tat peptides linked to Biotin was found to associate with SETDB1, SETDB2, and SUV39H1 when using whole cell extracts (Figure 1A, Lane 3). An acetylated Tat peptide (lysine residues 50, 51) linked to Biotin was used as a test for specificity of the enzyme binding *in vitro *(Figure 1A, Lane 4). SUV39H1 was present in the complex with the unmodified and the acetylated Tat peptides; however SETDB1 and SETDB2 exhibited specificity for only the unmodified Tat peptide. Figure 1B utilized the same pull-down complexes with Biotin-labeled wild type and acetylated Tat and probed for the presence of G9a and SETMAR. Both methyltransferases were found to associate with the wild type and acetylated forms of Tat (although less binding with SETMAR), therefore not conferring specificity for the modifications tested (Lanes 2 and 3). We then asked if the binding of SETDB1 to wild type Tat was specific using Westerns for BRG1 as well as performing Tat peptide and protein competitions. We have previously shown that acetylated Tat has a high affinity for bromodomain-containing complexes including members of the SWI/SNF family [61,62]. Results in panel C show that acetylated Tat, but not unmodified Tat, bound to BRG1. We next performed peptide competition assays with the Tat 42–51 peptide (1:10 ratio) as well as using purified Tat 1–86 (1:10 ratio) and found a complete competition when assaying for the presence of SETDB1.

As SETDB1 and SETDB2 were found to bind the unmodified Tat peptide, we next looked at the interaction with the full length wild type Tat protein. GST-bound Tat and Tax (control) proteins were allowed to incubate with whole cell extracts, and the associated complexes were probed for the presence of SETDB1 and SETDB2. SETDB1 was shown to associate with the full length Tat protein in greater abundance than SETDB2 (Figure 1D, Lane 3). The results of panels A-D are summarized in Figure 1E. Here, each enzyme utilized in our *in vitro *binding assay is depicted for their Tat binding affinity indicated on the right-hand side. SETDB1 and SETDB2 have the greatest affinity for wild type Tat, whereas, SUV39H1, SUV39H2, G9a, and SETMAR all bound to both unmodified and acetylated Tat to varying degrees. As SETDB1 had the highest affinity over SETDB2, this enzyme became the focus of further experimentation.

### SETDB1 knockdown increases the transactivation of the viral LTR

**Figure 2:**
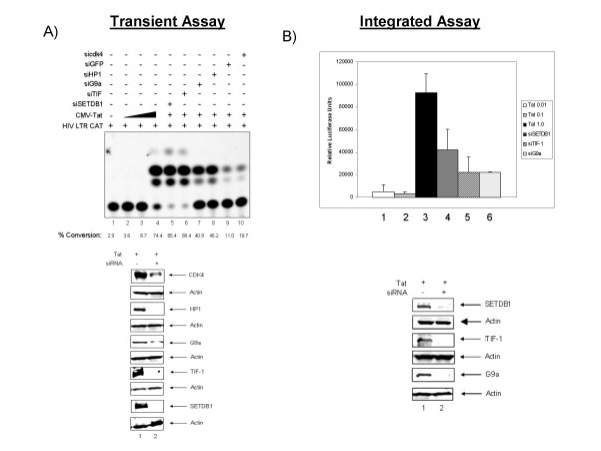
**Transiently transfected and integrated LTR reporter systems exhibit increased transactivation in the absence of SETDB1**. **A) **Transient transfection of the CAT assay is broken down as follows: Lane 1 indicates the negative control; Lanes 2–4 titration of Tat from 0.01, 0.1 and 1.0 ug to establish a range of activation; Lanes 5–10 are in the presence of 0.1 ug Tat as well as the indicated transfected siRNAs. **B) **TZM-bl cells containing an integrated LTR-Luc were transfected with siGFP, siSETDB1, siTIF-1, siG9a, and siHP1 in addition to Tat (0.1 ug) to initiate transcription. Confirmation of the knockdown of SETDB1 is shown in a Western blot below. Each transfection and luciferase assay was repeated at least three times.

Results above indicated that SETDB1 may be a potential candidate for the methylation of Tat. Next, we performed two tandem experiments; one which utilized a transient transfection of the LTR-CAT reporter system and one that utilized an integrated LTR-Luc reporter system. We performed a LTR CAT transfection experiment with increasing amounts of Tat and various fixed concentrations of siRNAs against SETDB1 and other related enzymes. We also used siGFP and siCDK4 as two negative controls in the transfection. Results in Figure 2A indicate that LTR activity is low at 3 ug concentration in CEM cells (panel A, lane 1) while increasing concentrations of Tat increased the activated transcription (0.01, 0.1, 1.0 ug; lanes 2 – 4). The LTR activity was maximal in the presence of 1.0 ug of Tat in these assays. We then asked if siRNAs against various methyltransferases could indeed activate the LTR in the presence of suboptimal concentrations of Tat. Results of such an experiment are shown in Figure 2A lanes 5 – 10. All of these lanes were transfected with LTR CAT at 1.0 ug and Tat at 0.1 ug per transfection. This low concentration of Tat normally does not optimally activate LTR transcription in these cells as seen in lane 3. Results of siRNA transfections indicate that suppression of SETDB1 and TIF-1 show the maximal amount of activity, followed by G9A and HP1. Surprisingly, the two controls, i.e. siGFP and siCDK4, also showed somewhat of an increase transcriptional activity, thereby serving as negative controls for siRNA transfection. None of these siRNAs activated the basal transcription of LTR (data not shown). All four siRNAs against SETDB1, TIF, G9A, and HP1 decreased the endogenous protein levels by more than 80% (the bottom of panel A).

We next performed a similar set of experiments in an LTR integrated system. TZM-bl cells are HeLa cells which contain both an integrated LTR-Luc reporter gene and an integrated LTR-β-Gal gene. To initiate viral transactivation, Tat must be transfected into these cells. We plated cells and allowed them to grow overnight before transfecting both Tat and the relevant siRNAs. We initially titrated Tat at 0.01, 0.1, and 1.0 ug to ensure that we could obtain an accurate standard curve for the luciferase readings (data not shown). Next, we transfected Tat into the cells at 0.1 ug, a suboptimal level, so that we could detect subtle differences in transcription activity resulting from the siRNA knockdowns. siGFP, siSETDB1, siTIF-1, and siG9a were all transfected along with Tat and 48 hours later cells were harvested for a luciferase assay. Figure 2B shows the results of the luciferase assay with the each value normalized to the siGFP control and activation represented in relative luciferase units. The knockdown of SETDB1 in these cells resulted in ~12 fold increase in activation as compared to the Tat control alone (lane 2). The knockdown of the other two proteins resulted in about ~6 fold increase in activation as compared to the Tat control. A confirmation western blot of the knockdown of SETDB1 and other proteins are shown on the bottom of panel B. Collectively, these results imply that reduced SETDB1 levels in a cell results in greater activation of the LTR.

### Methylation of Tat at Lysines 50 and 51 by SETDB1 and their functional significance

**Figure 3:**
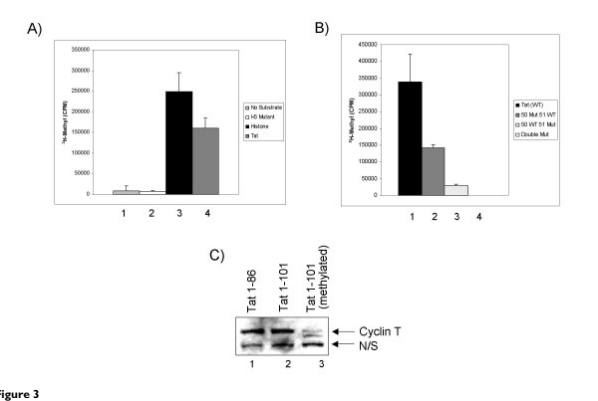
***In vitro *methyltransferase assays with SETDB1 reveal preferential methylation of Tat lysine 51 and loss binding to cyclin T**. **A) **The panel contains the negative and positive controls for the methylation assay. Both "no substrate" and histone H3 N-terminal mutant (K to A at positions 4, 9, 14, 18, 23, 27, 36, and 37) serve as negative controls. Wild type Tat 1–86 protein was used for *in vitro *methylation assay. **B) **The panel shows the incorporation of *methyl*-^3^H onto the Tat mutant peptides. Tat K50A showed a ~2 fold drop in counts, whereas the K51A showed more than ~10 fold drop in activity. **C) **Purified biotin labeled TAR RNA or PolyU RNA was mixed with purified proteins including wild type Tat 1–86, Tat 101, methylated Tat 101, purified Cdk9/cyclin T (data not shown) or extract. Unmodified and methylated Tat (1–86 and 1–101) were incubated with CEM nuclear extract containing endogenous Cdk9/cyclin T complexes (both active and inactive small and large complexes). Biotin-TAR RNA was added to the reaction mixture at the same time, processed and western blotted for presence of cyclin T.

Next, we asked which lysine residues could specifically be methylated by SETDB1. We utilized an *in vitro *methyltransferase assay incorporating a reaction mixture containing substrate, enzyme, buffer, and *S*-Adenosyl-L-[*methyl*-^3^H] methionine as a source of radio-labeled methyl groups. Purified SETDB1 enzyme was incubated with either no substrate, histone H3 N-terminal peptide mutated at all 8 lysines (residues 2–37), four core histones or WT Tat protein as a control as well as Tat mutant peptides: K50A, K51A, and K5051A. The reaction mixtures were incubated overnight at 37°C, spotted on GF/C filters and washed to remove any free radioactivity. The filters were then added to scintillation vials and counts were taken. Figure 3A summarizes the results of the controls, confirming that the enzyme was active when using full length Tat or core histones with multiple lysine residues. Both "no substrate" and Histone H3 mutant peptide showed very minimal background counts. Interestingly the level of Tat methylation using SETDB1 enzyme *in vitro *was far more efficient as compared to the 4 core histones that normally contained more than 20 lysine residues in both the N-terminus and the core domains of histones. Next, we utilized wild type and Tat peptide mutants to further define the residues that are methylated in Tat. Figure 3B summarizes the experimental results for each of the Tat peptide mutants. Overall we observed a two fold drop in activity when using a K50A mutant, whereas there was more than a 10 fold drop when using the K51A mutant peptide. Double mutant peptide at lysines 50 and 51 showed no methylation activity. Collectively, these results imply that both Tat lysine 50 and 51 are methylated, however lysine 51 is much more efficiently methylated when using SETDB1 as the enzyme. Finally, it is important to note that we have not been able to conclusively determine whether lysine 51 is either mono- di- or tri- methylated (although we have observed tri-methylation of Tat in IP experiments, data not shown) hence a possible reason for the better labeling of lysine 51 results seen *in vitro*.

We next asked whether methylation of Tat alters the specificity of cyclin T/TAR RNA binding *in vitro*. To do that, we used a biotin TAR pull-down RNA experiment and asked whether wild type or methylated Tat could still bring down cyclin T. Our initial set of experiments showed that when the reaction mixture contained TAR RNA (but not Poly-U RNA), wild type Tat, and purified Cdk9/cyclin T complex the affinity of cyclin T to TAR was fairly stable (data not shown). Next, we incubated purified methylated Tat 101 protein with TAR RNA and extract from CEM T-cells that contained endogenous Cdk9/cyclin T complexes. Following incubation and pull-down of TAR associated complexes, samples were separated on a 4–20% gel and Western blotted for the presence of cyclin T. Results, in Figure 3C showed that both unmodified Tat 86 or Tat 101 were able to bind to TAR RNA (lanes 1 and 2). However, methylated Tat was unable to form a Tat/cyclin T/TAR ternary complex *in vitro *(lane 3). Collectively, these results indicate that Tat methylation may decrease the affinity of Cdk9/cyclin T to the TAR RNA molecule.

### Effect of siSETDB1 on HIV-1 reactivation

**Figure 4:**
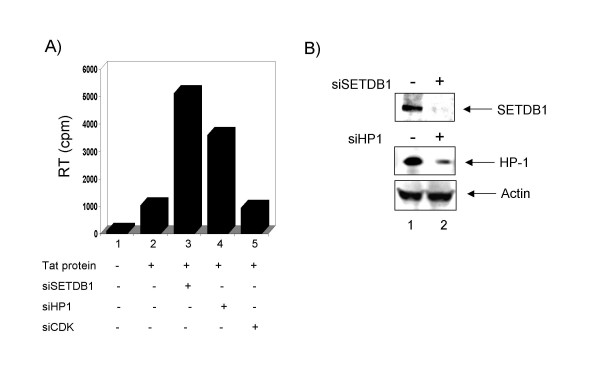
**Effect of siSETDB1 on HIV-1 progeny formation**. Log phase HLM-1 cells were electroporated with siSETDB1 and siHP1 for 48 hrs. Cells were subsequently removed and incubated with Tat for 4 hrs at 37°C in RPMI without serum. Cells were then plated in complete media for 6 days at 37°C and supernatants were process for RT activity. **A) **The effect of purified Tat protein on HLM-1 activation (lane 2) and subsequent super-activation with siSETDB1 and Tat protein in HLM-1 cells (lane 3). Lane 4 was with siHP1 and lane 5 with siCDK scrambled RNA. **B) **Western blot of transfected cells for SETDB1, HP1 and actin. Cell extracts were processed post siRNA transfection and western blotted for various proteins. For the actin westerns, Lane 1 is from siSETDB1 treatment and lane 2 is from siHP1 treatment.

We finally asked if suppression of SETDB1 could indeed activate a latent virus. For this purpose we transfected HLM-1 cells with two siRNAs, siSETDB1 and siHP1. HLM-1 cells are Hela T4 cells that contain one copy of mutated virus in the Tat region (triple termination codon). These cells could be used to activate virus with Tat or various other stimuli including TNF. We therefore used siSETDB1 and siHP1 to first transfect HLM-1 cells and incubated samples at 37°C for 48 hrs. We then removed cells from the plate and incubated them with Tat protein for 4 hrs at 37°C. Subsequently, cells were plated again in complete media. Tat has the ability to go through the cellular membrane and activate HIV-1 LTR when incubated with cells. Samples were carried out for 6 days and supernatants were processed for RT activity. As seen in Figure 4A, addition of no Tat showed no RT activity (Lane 1) however, Tat protein was able to activate the virus after 6 days (Lane 2). The efficiency of viral production is usually low with the addition of just Tat to the cells in the absence of any other manipulations. Cells treated with siSETDB1 (Lane 3) and siHP1 (Lane 4) showed activation of the virus, but not siCDK2 scramble (Lane 5). The levels of SETDB1 and HP1 were reduced in these transfected cells as judged by the Western blot in Panel B. Collectively, these results further imply that SETDB1 suppression is mediating a better activated transcription and viral progeny formation.

## Discussion

We have previously shown that acetylation of Tat lysine residues 50 and 51 results in an increase in transactivation of the LTR and promotes the incorporation of the Cdk9/cyclin T complex as well as other transcription factors into the active complex [52]. As acetylation serves as an activation signal for Tat, it is safe to suggest that there is also a counter regulatory repression signal [63,64]. Indeed, very recently Boulanger *et al. *and Xie *et al. *have shown that the methylation of Tat arginine residues 52 and 53 result in a decrease in association with viral transcription factors, as well as compromised transcriptional activation of the LTR [54,60]. Here we propose that the methylation of Tat lysine 50 and 51 can result in a decrease in viral transcription.

The post-translational modifications observed on the histone tails can be easily correlated to modifications observed on other proteins. Commonly seen trends of modifications arise such as acetylation as a marker for activation (i.e. the transition from heterochromatin to euchromatin to initiate transcription) and methylation as a marker for repression (i.e. the addition of methyl groups to DNA to silence gene expression). Interestingly, the amino acid residues that can usually accept a post-translational modification are less frequent throughout a protein, but are also usually involved in key interactions, whether it can maintain the tertiary structure, enzymatic active sites, or binding sites for protein-protein interactions.

We show here that the lysine residues of Tat which are prone to acetylation, 50 and 51, can be preferentially methylated *in vitro *by the histone methyltransferase SETDB1. We show that the knockdown of this enzyme causes an increase in the transactivation of the viral LTR. The siRNA transfection experiments also included siRNAs against TIF1, G9a, and HP1. SETDB1 as a histone methyltransferase trimethylates H3K9, therefore initiating the formation of heterochromatin and gene silencing [65]. This H3K9 methylation also serves as a mark for recruitment of the HP1 family of heterochromatin proteins [66]. Therefore, it is possible that the methylation of Tat by SETDB1 could recruit HP1 and initiate transcriptional silencing through chromatin remodeling.

We have previously shown that Tat binds to a number of critical proteins including pCAF, Cyclin T1, and TIF-1 [31]. TIF-1α is a member of the TRIM (tripartite motif) family of proteins. TRIM proteins contain the TRIM domain which is composed of three zinc-binding domains, a RING, a B-box type 1, and a B-box type 2, followed by a coiled-coil region. The TRIM domain mediates protein-protein interactions [67] and oligomerization [68]. TIF-1α has been demonstrated to be a repressor of RXR nuclear hormone receptors [69]. TIF-1 (TRIM24) exhibits sequence similarities with the HIV restriction factor, TRIM5α, including the TRIM domain. It would be intriguing to find out if TIF-1 controls similar pathways as TRIM5α and could be a possible restriction factor for HIV-1 gene expression or control of methylation of nucleic acids. Possible reasoning for this is that TIF-1α has been shown to bind to HP1α, HP1β, TFIIE, Hsp70, PML, TAFII55, Zinc finger protein 10, RAR alpha, TAFII28, THR alpha 1, and other TIF-1 subunits.

siRNA mediated knock-down of various HMTs, including TIF-1 and SETDB1, indicated that decreased methyltransferase activity increased HIV LTR transcription in transient transfection assays. We also showed that the methylation of Tat by SETDB1 is preferential for both lysines 50 and 51. It is possible that any of these proteins is being mono-, di-, or tri- methylated by SETDB1 at any given time "on" or "off" of the HIV-1 LTR. Therefore, future experiments will determine the rate and type of Tat methylation on the LTR and in the presence of TAR RNA.

**Figure 5:**
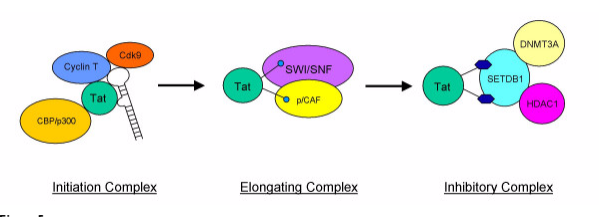
**The proposed model for the interaction of Tat with SETDB1 and chromatin remodelers in HIV-1 transcription**. This model depicts the role of Tat in the involvement of activating transcription and chromatin remodeling. Tat is shown interacting with Cdk9/cyclinT to bind to the TAR secondary structure element to initiate transcription. This binding complex recruits CBP/p300 which acetylates Tat, dissociates from the complex, and associates with SWI/SNF and p/CAF to facilitate transcriptional elongation. The repressive complex is shown with Tat being methylated by SETDB1, which may interact with DNA methyltransferase 3A and recruits HDAC to promote a compacted heterochromatin structure possibly at the 3' end of the HIV-1 genome.

Although we have shown that the lysine 51 of HIV-1 Tat can be methylated by SETDB1, it is unlikely that this modification alone completely shuts down the promoter activity. We propose that the interaction of SETDB1 with Tat methylates the protein and that may be responsible for the recruitment of part of the transcriptional repression machinery to the HIV-1 genome. Figure 5 depicts our current model for the initiation, elongation, and repression of the promoter in relation to Tat modifications. The first scenario predicts that unmodified Tat initiates transcription by binding to TAR and recruiting the pTEFb into the active complex. This leads to the acetylation of Tat by CBP/p300. The second scenario promotes the elongation of transcription by complexing with various other transcription factors including remodeling complexes such as SWI/SNF and p/CAF. The third and last step proposes that Tat is methylated by SETDB1 and the enzyme recruits DNA methyltransferase 3A (DNMT3A) and HDAC to the elongation complex (possibly toward the 3' end of the HIV-1 genome) to repress transcription and promote heterochromatin formation. SETDB1 has previously been shown to directly interact with DNMT3A to promote gene silencing [70] and it has also been shown to interact with HDAC [71] which promotes the deacetylation of histones and formation of heterochromatin. The recruitment of these gene silencing proteins to the HIV-1 genome by the methylation of Tat may be a strong indication for a possible transcriptional repression of the LTR. Future experiments using ChIP assays will determine if such complexes do indeed exist as the 3' end of the HIV-1 genome after active transcription has occurred and prior to mRNA translation, packaging, and release of the virus.

## Materials and methods

### SiRNA and protein Reagents

Control and SETDB1, HP1-γ, TIF-1α, and G9a double stranded RNA oligonucleotides (siRNA) were purchased from Dharmacon Research (Lafayette, CO). Human SETDB1 and human SETDB2 proteins were expressed in baculovirus infected insect cells as amino-terminal fusion proteins with poly-histidine (H6) or H6-maltose binding protein (H6MBP). Baculovirus constructs were generated by Gateway recombinational cloning of cDNA clone, KG1T for *SETDB1*, (a generous gift from Dr. Greg Matera, Case Western Reserve University) and I.M.A.G.E. clone 5266911 for *SETDB2 *(Open Biosystems). Proteins were purified from soluble extracts by immobilized metal affinity chromatography (IMAC) using a nickel charged HisTrap-HP prepacked column (GE Healthcare) followed by anionic exchange using a HiTrap Q prepacked column (GE Healthcare) (H6MBP-SETDB1 only). Proteins were stored in buffer containing 20 mM Tris-HCl pH8.0, 50 mM NaCl, 10% glycerol, and 1 mM dithiothreitol at -80°C. Protein concentration was determined by Bradford assay (BioRad) relative to BSA.

Core human histones (all four) were purified from Hela cells and WT Tat 1–86 was overexpressed in an *E. coli *system followed by column purification [72]. Anti-ESET(SETDB1) and anti-SUV39H1 antibodies were purchased from Cell Signaling (Danvers, MA). Anti-SETDB2 antibody was purchased from Abgent (San Diego, CA). Anti-SETMAR and anti-G9a antibodies were purchased from Abcam (Cambridge, MA). Tat WT and mutant peptides were synthesized and purchased commercially from SynBioSci (Livermore, CA) with the following sequences: Tat WT 45–54 (I-S-Y-G-R-K-K-R-R-Q), Tat K50A (I-S-Y-G-R-A-K-R-R-Q), Tat K51A (I-S-Y-G-R-K-A-R-R-Q), Tat K50, 51A (I-S-Y-G-R-A-A-R-R-Q). The purity of each peptide was analyzed by HPLC to greater than 98%. Mass spectral analysis was also performed to confirm the identity of each peptide as compared to the theoretical mass (Applied Biosystems Voyager System 1042). Peptides were resuspended in dH2O to a concentration of 1 mg/mL. Biotin-Tat and Biotin-Acetylated Tat were purified as published previously [52].

### Cell Culture

C8166 is an HTLV-1 infected T-cell line and TZM-bl is a cell line derived from HeLa cells containing Tat-inducible Luciferase and β-Gal reporter genes. C81 cells are grown in RPMI-1640 media containing 10% FBS, 1% L-glutamine, and 1% streptomycin/penicillin (Quality Biological). TZM-bl cells were grown in Dulbecco's modified Eagle's medium (DMEM) containing 10% FBS, 1% L-glutamine, and 1% streptomycin/penicillin (Quality Biological). All cells were incubated at 37°C and 5% CO_2_. Cells were cultured to confluency and pelleted at 4°C for 15 min at 3,000 rpm. The cell pellets were washed twice with 25 mL of phosphate buffered saline (PBS) with Ca^2+ ^and Mg^2+ ^(Quality Biological) and centrifuged once more. Cell pellets were resuspended in lysis buffer (50 mM Tris-HCl, pH 7.5, 120 mM NaCl, 5 mM EDTA, 0.5% NP-40, 50 mM NaF, 0.2 mM Na_3_VO_4_, 1 mM DTT, one complete protease cocktail tablet/50 mL) and incubated on ice for 20 min, with a gently vortexing every 5 min. Cell lysates were transferred to eppendorf tubes and were centrifuged at 10,000 rpm for 10 min. Supernatants were transferred to a fresh tube where protein concentrations were determined using Bio-Rad protein assay (Bio-Rad, Hercules, CA).

### siRNA Transfection

SETDB1-directed siRNA pool (ON-TARGET *plus SMART*pool reagent L-020070-00), TIF-1α-directed siRNA pool (ON-TARGET *plus SMART*pool reagent L-005387-00), HP1-γ-directed siRNA pool (ON-TARGET *plus SMART*pool reagent L-010033-00) and G9a-directed siRNA pool (ON-TARGET *plus SMART*pool reagent L-006937-00) were purchased from Dharmacon. TZM-bl cells were seeded in 6 well plates at 400,000 cells/well in DMEM containing 10% FBS. The following day, the cells were transfected with 0.01, 0.1, or 1.0 ug Tat plasmid and/or with either siGFP, siSETDB1, siTIF-1, siG9a, or siHP1-γ (Dharmacon) using Metafectene (Biontex) lipid reagent. Total amount of siRNA was held constant using siGFP. Cells were harvested forty-eight hours post transfection for protein concentration and luciferase readings.

### Biotin-Tat Pull-Down

Tat peptides (amino acids [aa] 42 to 52) were synthesized with a biotin tag on a PAL-polyethylene glycol-polystyrene resin by continuous flow solid-phase synthesis on a Perspective Biosystems Pioneer synthesizer (Framingham, MA) using HBTU-activated 9-fluorenylmethoxy carboxyl amino acids and were synthetically acetylated at positions 41/50/51 or 50/51, respectively [52]. Synthesized Tat peptides (aa 36 to 53 and 42 to 54), labeled with biotin at the N terminus and with or without an acetyl group at lysines 50 and 51, were used in the pull-down assays. C81 whole cell extracts (2 mg) were prepared and incubated with biotin labeled Tat peptides (WT and acetylated, 1.0 ug) in TNE_50 _buffer (100 mM Tris-HCl, pH 7.5; 50 mM NaCl; 1 mM EDTA; 0.1% NP-40) overnight at 4°C. Streptavidin beads (Boehringer Mannheim) were added to the mixture and incubated for 2 h at 4°C. The beads were washed once with each TNE_300, _TNE_150_, and TNE_50 _+ 0.1% NP-40. Bound proteins were separated on 4–20% SDS-PAGE gel and subjected to Western blotting with antibodies against SUV39H1, SUV39H2, G9a, SETDB1, SETDB2, and SETMAR.

### GST Pulldown

C81 whole cell extracts (2 mg) were prepared and incubated with 10 ug of purified GST-Tat and GST-Tax constructs in TNE_50 _buffer (100 mM Tris-HCl, pH 7.5; 50 mM NaCl; 1 mM EDTA; 0.1% NP-40) overnight at 4°C. The following day, a 30% Protein A & G bead slurry (CalBioChem, La Jolla, CA) was added to each reaction tube and incubated for 2 hours at 4°C. Samples were spun and washed twice with TNE_300 _+ 0.1% NP-40 (100 mM Tris, pH 8.0; 300 mM NaCl; 1 mM EDTA, 0.1% Nonidet P-40) and 1× with TNE_50 _+ 0.1% NP-40 to remove nonspecifically bound proteins. Samples were loaded and run on a 4–20% Tris-Glycine SDS-PAGE gel and subjected to Western blotting with antibodies against ESET/SETDB1 and SETDB2.

### TAR RNA Streptavidin bead pull-down assay

Purified biotin labeled TAR RNA (N terminus, 3 ug) or PolyU RNA were mixed with various purified proteins including wild type Tat 1–86 (0.5 ug), Tat mutant K50/51A (0.5 ug) or Baculovirus purified Cdk9/cyclin T (0.75 ug). Samples were incubated in TNE_50 _buffer (100 mM Tris-HCl, pH 7.5; 50 mM NaCl; 1 mM EDTA; 0.1% NP-40) with protease inhibitors and RNAsin overnight at 4°C. Streptavidin spharose beads (1/10 volume of a 30% slurry; Boehringer Mannheim) were added to the mixture and incubated for 2 h at 4°C. Bound proteins were separated on 4 to 20% sodium dodecyl sulfate – polyacrylamide gel electrophoresis (SDS-PAGE), and subjected to Western blotting with anti-cyclin T antibody.

GST-Tat 101 protein (2 mg) was first labeled *in vitro *with purified SETDB1 and *S*-Adenosyl-L-[*methyl*-^3^H] methionine. The reaction was incubated overnight at final volume of 35 ul. Also, 35 ul of sterile mineral oil was added to top of reaction to avoid evaporation of the reaction during the overnight incubation. The next day, 15 ul of 30% Glutathion beads were added for 2 hrs at 4°C and unbound material was washed with TNE_50 _+ 0.1% NP-40. GST-Tat protein was eluted for 4 hrs at 37°C with reduced Glutathione. Purified methylated Tat was next incubated with CEM nuclear extract containing endogenous Cdk9/cyclin T complex (both active and inactive complex) at a final 2 mg/reaction. Biotin-TAR RNA at 1.5 ug was also added to the reaction mixture at the same time. Samples were incubated in TNE_50 _buffer with protease inhibitors and RNAsin overnight at 4°C. Subsequent reaction procedures were similar to what was described above.

### *In vitro *methyltransferase and Filter Binding Assay

Full length WT Tat (3 ug), Tat peptides (2 ug), Tat mutant peptides (2 ug), histone H3 mutant peptide (2 ug, K to A mutations at residues 4, 9, 14, 18, 23, 27, 36, and 37) and core histones (1 ug) were incubated with 2 μg of purified enzyme (SETDB1, SETDB2) in the presence of 0.55 μCi *S*-Adenosyl-L-[*methyl*-^3^H] methionine (GE Healthcare, Piscataway, NJ) and reaction buffer (50 mM Tris-HCl, pH 8.5, 20 mM KCl, 10 mM MgCl_2_, 250 mM sucrose, 10 μM β-mercaptoethanol) overnight at 37°C in a final reaction volume of 30 μl. The overnight methylation reactions were spun briefly and spotted on GF/C membranes (Millipore) in duplicate and allowed to dry. The filters were washed three times in excess cold 10% TCA, 1% sodium phosphate followed by once with 100% ethanol. The filters were allowed to dry and counted in Beckman Coulter LS6001C scintillation counter in scintillation fluid.

### Transfection of HLM-1 cells

Log phase HLM-1 cells (5 × 10^6^/sample) were electroporated (210 volts, 800 mA) with siSETDB1 and siHP1 and incubated in complete media for 48 hrs. Cells were subsequently washed and treated with tyrpsin for 2 min. Next, cells were washed and incubated with Tat (10 ug) in Tat buffer (PBS + 0.01 mM DTT) for 4 hrs at 37°C in RPMI without serum. Cells were then plated in complete media for 6 days at 37°C and supernatants were process for RT activity.

### Luciferase Assay

Forty-eight hours post transfection, luciferase activity of the firefly luciferase of the TZM-bl cells was measured with the DualGlo Luciferase Assay (Promega). Luminescence was read from a 96 well plate on an EG&G Berthold luminometer. LTR driven firefly luciferase levels were normalized to siGFP levels. Data shown represents at least two repeats of each condition.

### CAT Assay

Plasmids (LTR-CAT or CMV-Tat) were transfected by electroporation using a Bio-Rad Gene Pulser (Bio-Rad, Richmond, CA) at 960 μF and 230 Volts. After 48 h, cells were lysed and chloramphenicol acetyltransferase (CAT) and luciferase activities were determined. Luciferase was measured using the Luciferase assay system (Promega). For the CAT assay, a standard reaction was performed by adding the cofactor acetyl coenzyme A to a microcentrifuge tube containing cell extract (50 ug) and radiolabeled (^14^C) chloramphenicol in a final volume of 50 μl and incubating the mixture at 37°C for 1 h. The reaction mixture was then extracted with ethyl acetate and separated by thin-later chromatography on silica gel plates (Baker-flex silica gel thin-later chromatography plates) in a chloroform-methanol (19:1) solvent. The resolved reaction products were then detected by exposing the plate to a PhosphorImager cassette.

## Competing interests

The authors declare that they have no competing interests.

## Authors' contributions

RVD performed the Biotin Tat pulldowns, the LTR-Luc and siRNA transfections, the luciferase assays, and the *in vitro *methyltransferase assays, RE performed the siSETDB1 confirmatory Westerns, the Biotin-TAR pull-down, and cyclin T1 Westerns, WW performed the GST-Tat/Tax pull-downs, RB provided the Biotin-Tat reagents and assisted with the pulldowns, ZK provided the TZMbl cells and assisted with the luciferase assays, EF and DS provided the purified SETDB1 and SETDB2 enzymes, WW, KKH, and FK participated in the design and discussion of the study. All authors read and approved the manuscript
